# *Ixeridium calcicola* (Compositae), a New Limestone Endemic from Taiwan, with Notes on Its Atypical Basic Chromosome Number, Phylogenetic Affinities, and a Limestone Refugium Hypothesis

**DOI:** 10.1371/journal.pone.0109797

**Published:** 2014-10-08

**Authors:** Koh Nakamura, Shih-Wen Chung, Yoshiko Kono, Meng-Jung Ho, Tian-Chuan Hsu, Ching-I Peng

**Affiliations:** 1 Biodiversity Research Center, Academia Sinica, Nangang, Taipei, Taiwan; 2 Botanical Garden Division, Taiwan Forestry Research Institute, Taipei, Taiwan; Field Museum of Natural History, United States of America

## Abstract

A new species *Ixeridium calcicola* (Compositae) endemic to middle altitude (ca 1,000–2,000 m asl) limestone mountains of eastcentral Taiwan is described based on morphological and chromosome cytological observations and molecular phylogenetic analyses. *Ixeridium calcicola* resembles *Ixeridium transnokoense*, endemic to upper montane and alpine ranges (2,600–3,500 m asl) of Taiwan, in the dwarf habit, but differs in the oblong to lanceolate leaf blades (vs. linear to linear-lanceolate), the presence of mucronulate teeth on the leaf margin and petiole (vs. smooth to very sparse), the dark purple lower leaf surface (vs. greenish), the capitulum with 10 to 12 florets (vs. 5 to 7) and 8 to 10 inner phyllaries (vs. 5, rarely to 7). The basic chromosome number in *Ixeridium* was known as X = 7. However, the new species has a basic chromosome number of X = 8, as recorded also in the closely related *Ixeris*. Molecular phylogenetic analyses with the expanded sampling of *Ixeridium* and *Ixeris* including both type species supported the monophyly of each of the genera and the placement of the new species in *Ixeridium*. The result of the phylogenetic analyses and detailed observation of the chromosome morphology revealed that X = 8 in *Ixeridium calcicola* is derived from centric fission in an ancestral karyomorphotype with X = 7 in *Ixeridium*. *Ixeridium calcicola* and *Ixeridium transnokoense* formed a Taiwan endemic lineage and their estimated divergence time was in the middle Pleistocene. Their common ancestral lineage may have experienced altitudinal distribution shifts in response to glacial-interglacial temperature fluctuation, and a lineage which had not retreated to alpine ranges in an interglacial period likely survived in a limestone refugium, where ordinary plant species did not grow, leading to allopatric speciation.

## Introduction

Limestone environments harbor plant species adapted to highly alkaline and thin soils and desiccation on porous limestone bedrock [1], [2]. Because of the edaphic isolation from surrounding environments and the fragmented distribution of limestone outcrops, many limestone plants are local-endemic or, in extreme but not rare cases, site-endemic [3]. Species diversity of limestone floras is considered to be underestimated as a result of the difficulty of sampling in the rugged terrain, whereas limestone environments are increasingly threatened by modern destructive land uses such as limestone quarrying [1]. Limestone environments are among the first priority habitats for botanical exploration and activities for species diversity conservation. In Taiwan Island, the distribution of limestone is restricted; metamorphosed limestone (or marble) is found in the eastcentral part and limestone of raised coral reefs is in small coastal areas of the southwestern and southernmost parts [4]. Many plant species of various families have been described from both types of limestone environments including very recent discoveries [5], [6], [7], suggesting that limestone areas in Taiwan are still not fully explored.

**Figure 1:**
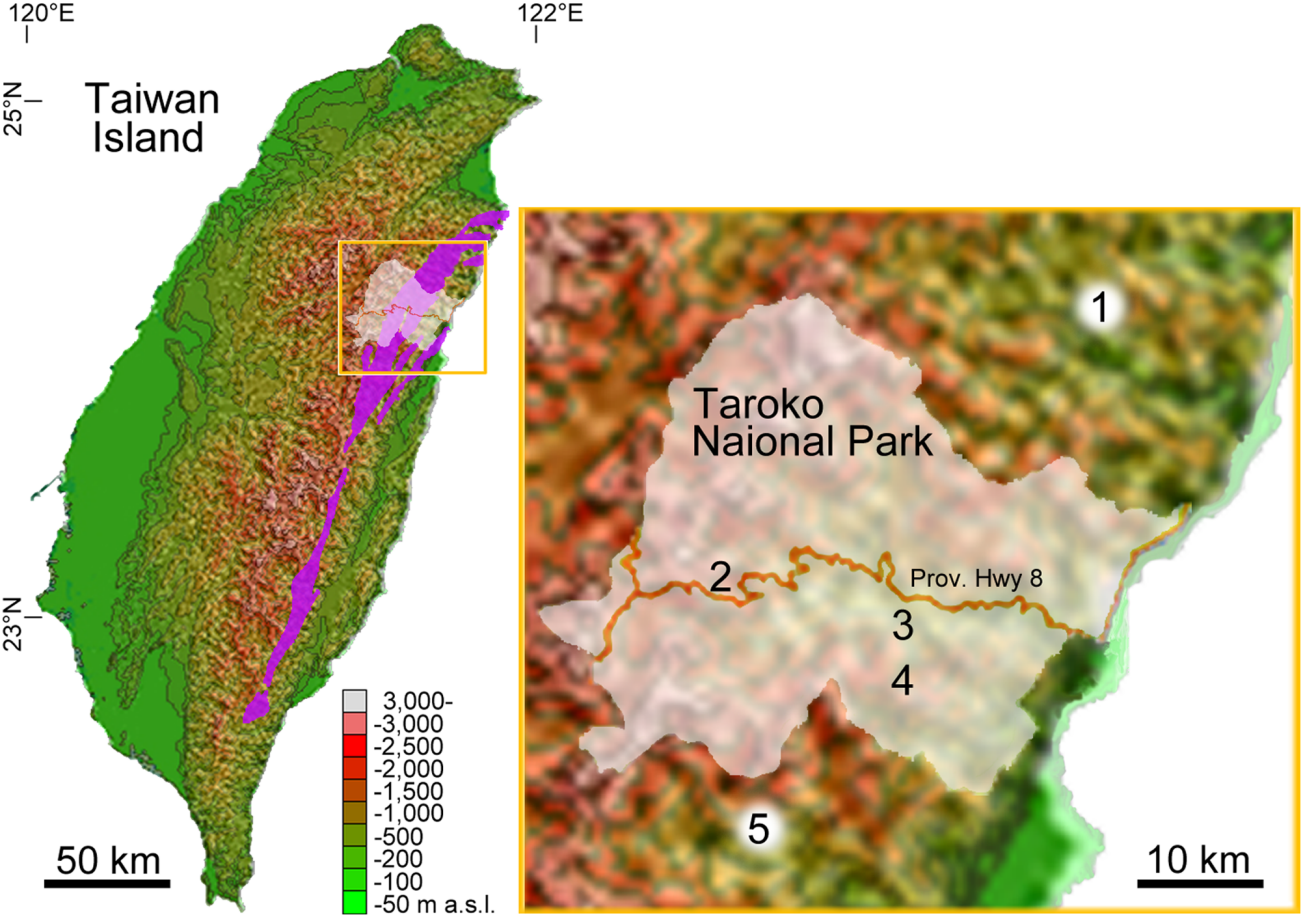
Localities of the new species *Ixeridium calcicola* in Taroko National Park and the vicinities, eastcentral Taiwan. 1. Hopin forest trail; 2. Pilu; 3. Yenhai Forest Road, 4. Luanshan to Potolushan, 5. Tien-tsang Cliff. White-shaded area indicates Taroko National Park and purple-shaded areas show the distribution of metamorphosed limestone.

Taroko National Park in eastcentral Taiwan (Fig. 1) contains Taroko Gorge and its surrounding area, which consists predominantly of relatively weak, intensely deformed marble and schist [8]. In our botanical survey at middle altitudes (ca 1,000–2,000 m asl) of limestone mountains in the national park and vicinities, an unknown species of *Ixeridium* (A.Gray) Tzvelev (Compositae, tribe Cichorieae) was discovered. *Ixeridium* comprises about 15 species predominantly in East and Southeast Asia [9], and in Taiwan the genus is represented by two species: *Ixeridium laevigatum* (Blume) Pak & Kawano and *Ixeridium transnokoense* (Sasaki) Pak & Kawano [10]. The unknown species resembled *Ixeridium transnokoense*, a plant of 10–20 cm tall endemic to the upper montane and alpine areas (2,600–3,500 m asl) of Taiwan Island occurring on grassy slopes [9], [10], in being a dwarf species. The other species *Ixeridium laevigatum* grows up to 90 cm tall and has the widest distribution among the congeners, ranging from East and Southeast Asia to New Guinea. It occurs from sea level to about 2,300 m asl but predominantly in lowlands and grows in various environments such as forest margins, grassy areas, riverbanks, cliffs, roadsides, and on gravelly limestone [9], [10]. After a close examination of living materials, herbarium specimens and literature, we came to the conclusion that the unknown plants represented an as yet undescribed species. However, our cytological study revealed that it has a basic chromosome number atypical for *Ixeridium* but diagnostic for an allied genus *Ixeris* (Cass.) Cass. of the same subtribe Crepidinae. *Ixeris* comprises about eight species in East and South Asia [11]. Both genera are rosulate herbs characterized by main axis bearing synflorescence (no lateral flowering stems), many capitula, long-beaked achenes, and persistent pappus of simple setose bristles (not feathery) [10], [12]. *Ixeridium* and *Ixeris* are separated based on the following morphological (especially carpological) and karyological features: number of florets per capitulum (5–12 in *Ixeridium*; 15–41 in *Ixeris*), achene morphology (ribbed with inconspicuous costa in *Ixeridium*; winged with well-developed costa and intercosta in *Ixeris*), pappus color (yellowish to straw-colored in *Ixeridium*; snow-white in *Ixeris*), and basic chromosome number (X = 7 in *Ixeridium*; X = 8 in *Ixeris*) [13], [14], [15]. However, there are a few exceptional *Ixeridium* species with a white pappus [14] and the number of florets may not differ markedly between some species of the two genera. In addition, such morphological features and basic chromosome number are known to have evolved in parallel in some genera of Crepidinae [16], [17]. As a result there remains a lack of consensus as to whether the two genera should be maintained [9], [10], [11], [12] or merged in *Ixeris* [18], [19], [20]. In previous molecular phylogenetic studies, species of the genera formed a clade within the subtribe but as both genera were represented by only one or two species their generic status could not be assessed [17], [21] and further molecular analyses with more extensive sampling are required.

The present study conducted detailed morphological and chromosome cytological observations and constructed a dated molecular phylogeny of *Ixeridium* and *Ixeris* with the expanded sampling including the type species of the two genera. We discussed the phylogenetic placement and taxonomic treatment of the new species, the evolution of the unusual chromosome number, and the origin of the new species.

## Materials and Methods

### Ethics statement

Sampling of the new species in Taroko National Park was conducted with the collect permits issued by the Headquarters of the national park.

### Nomenclature

The electronic version of this article in Portable Document Format (PDF) in a work with an ISSN or ISBN will represent a published work according to the International Code of Nomenclature for algae, fungi, and plants, and hence the new name contained in the electronic publication of a PLOS ONE article is effectively published under that Code from the electronic edition alone, so there is no longer any need to provide printed copies.

In addition, the new name contained in this work has been submitted to IPNI, from where the name will be made available to the Global Names Index. The IPNI LSIDs can be resolved and the associated information viewed through any standard web browser by appending the LSID contained in this publication to the prefix http://ipni.org/. The online version of this work is archived and available from the following digital repositories: PubMed Central, LOCKSS.

### Morphological observations

Living materials of the new species were used for morphological observations (*Shih-Wen Chung 11141, 11693*, TAIF and HAST). In addition, by checking herbarium specimens labelled as *Ixeridium transnokoense* in HAST, KYO, TAI, TAIF, TAIM, and TNM, we found a number of specimens representing the new species and used them. Specimens of genuine *Ixeridium transnokoense* in these herbaria (Appendix S1), including an isotype (*S. Sasaki s.n.*, KYO), were used for morphological comparison with the new species.

### Taxon sampling for molecular phylogenetic study

To test the generic assignment of the new species in *Ixeridium* and its phylogenetic distinction from congeners, the molecular analyses incorporated six out of eight species of *Ixeridium* in Taiwan and neighboring China, Japan, the Philippines, and Korea [9], [10], [11], [12], [18], [20], [22] and six out of eight species of *Ixeris* (excluding named hybrids) (Appendix S2). This included all the species of the two genera in Taiwan. Species not collected were all local endemics: *Ixeridium parvum* (Kitam.) Pak & Kawano in Yakushima Island of Japan [18], *Ixeridium yunnanense* C.Shih in Yunnan of China [9], and *Ixeris longirostra* Nakai in Ogasawara Islands of Japan [18]. The analyses excluded *Ixeridium aculeolatum* C.Shih and *Ixeridium sagittarioides* (C.B.Clarke) Pak & Kawano, which are considered to be misplaced in *Ixeridium* and are probably not members of Crepidinae [9]. The type species for both genera were included, namely *Ixeridium dentatum* (Thunb. ex Thunb.) Tzvelev and *Ixeris polycephala* Cass. Five of the six subspecies of *Ixeridium dentatum* [26] were also included. *Crepidiastrum lanceolatum* (Houtt.) Nakai, *Paraixeris denticulata* (Houtt.) Nakai, and *Youngia japonica* (L.) DC. were used as outgroups as these genera formed a sister clade of *Ixeridium–Ixeris* in a preceding study [21]. For the new species, *Ixeridium transnokoense*, and *Ixeridium laevigatum*, four, nine, and seven samples, respectively were collected from across the distribution ranges in Taiwan. For the other species one sample each was used. ITS sequences generated in this study (GenBank accession numbers AB972273–AB972301) and ITS sequences obtained from GenBank are denoted in Appendix S2.

### Molecular phylogenetic study

Molecular analyses hired the nuclear ribosomal internal transcribed spacer (ITS) region, which has been proven to be highly useful in resolving intra- and intergeneric relationships in Cichorieae in previous studies [17], [21], [23], [24], [25]. Total DNA extraction, polymerase chain reaction (PCR) amplification, and sequencing of ITS region (including ITS1 and ITS2 and the 5.8S rRNA gene) were conducted as described in [2]. Universal primers ITS1 and ITS4 [27] were used with the following PCR cycle conditions: 95°C for 5 min, 1 cycle of 97°C for 2 min, 50°C for 1 min, 72°C for 1 min, 25 cycles of 95°C for 1 min, 50°C for 2 min, 72°C for 3 min, and 72°C for 10 min. The same primers were used for sequencing. Cloning followed by sequencing was conducted for putative hybrids which showed double-peak nucleotide signals by direct sequencing, as described in [2]. DNA sequences were aligned using ClustalX ver. 1.8 [28] and then manually adjusted.

Bayesian phylogenetic and molecular dating analyses were conducted using BEAST ver. 1.7.5 [29], [30]. SYM+G substitution model was hired as the best-fitted model for the ITS data, as estimated using KAKUSAN4 [31]. The molecular clock hypothesis was rejected for the ITS data (*P*<0.0001) based on the likelihood ratio test [32], [33]. Therefore, a relaxed-clock uncorrelated lognormal distribution model was used for rate variation among lineages [34]. The Speciation Birth–Death Incomplete Sampling tree prior was employed for the branching rates [35]. The unweighted pair-group method of arithmetic averages (UPGMA) was used to construct a starting tree. Molecular dating hired previously published substitution rates for Cichorieae based on fossil calibrations, 8.7×10^−9^–10.8×10^−9^ substitutions site^−1^ year^−1^ [36], and applied the uniform-distribution prior in ucld.mean. The rates are slightly higher than generally reported rates for herbaceous plants with a minimum generation time of 1–3 years (minimum value = 1.72×10^−9^, mean value = 4.22×10^−9^, median = 3.72×10^−9^, maximum value = 8.34×10^−9^) [37], [38]. However, several lineages of temperate herbaceous Compositae have been reported to have higher ITS substitution rates based on calibrated phylogeny and the application of the higher ITS substitution rate have generated reasonable divergence time estimates in other Compositae genera [2], [39]. Default priors were used for the remaining parameters. MCMC chains were run for 50 million generations and sampled every 5,000 generations. Convergence of all parameters was checked using Tracer ver. 1.5.0 [40] and the first 1,000 of the 10,000 sampled trees were discarded as burn-in. The effective sample sizes of parameters in the log file were 1,988–37,058 after the burn-in, indicating satisfactory sampling of the posterior distributions of each parameter. A maximum clade credibility tree was estimated with a burn-in of 10% of the sampled trees and a posterior probability (PP) limit of 0.5 by TreeAnnotator ver. 1.5.4 [40], and visualized with FigTree ver. 1.3.1 [40].

Phylogenetic analysis was also conducted based on maximum parsimony (MP) criterion using PAUP* ver. 4.0b10 [33]. Indels were treated as missing data; scoring indels (except for length variation at mononucleotide repeats) as binary states following the simple indel coding strategy [41] gave the same topology (not shown). The characters were treated as unordered, and the character transformations were equally weighted. The branch collapse option was set to collapse at a minimum length of zero. A heuristic parsimony search was performed with 1,000 replicates of random additions of sequences with ACCTRAN character optimization, tree bisection*–*reconnection (TBR) branch swapping, and MULTREES and STEEPEST DESCENT options on. Statistical support for each clade was assessed by bootstrap analysis [42]. Ten thousand replicates of heuristic searches, with the TBR branch swapping option on and MULTREES options off, were performed to calculate bootstrap percentages (BP).

### Chromosome cytology

Somatic chromosomes were examined for the new species (*Ching-I Peng 23905*, HAST) and, for comparison, two congeners in Taiwan, *Ixeridium laevigatum* (*Ching-I Peng 23562*, HAST) and *Ixeridium transnokoense* (*Peng 23904*, HAST). Root tips were pre-treated in 2 mM 8-hydroxyquinoline solution at 15–18°C for 6–8 h, and then fixed overnight in a 31 ethanol-acetic acid solution below 4°C. Chromosomes were macerated and stained in 2% acetic orcein with 1 N hydrochloric acid (101) and observed. Classification of chromosome morphology was based on the position of centromere [43].

## Results

### Phylogenetic relationships based on ITS

**Figure 2:**
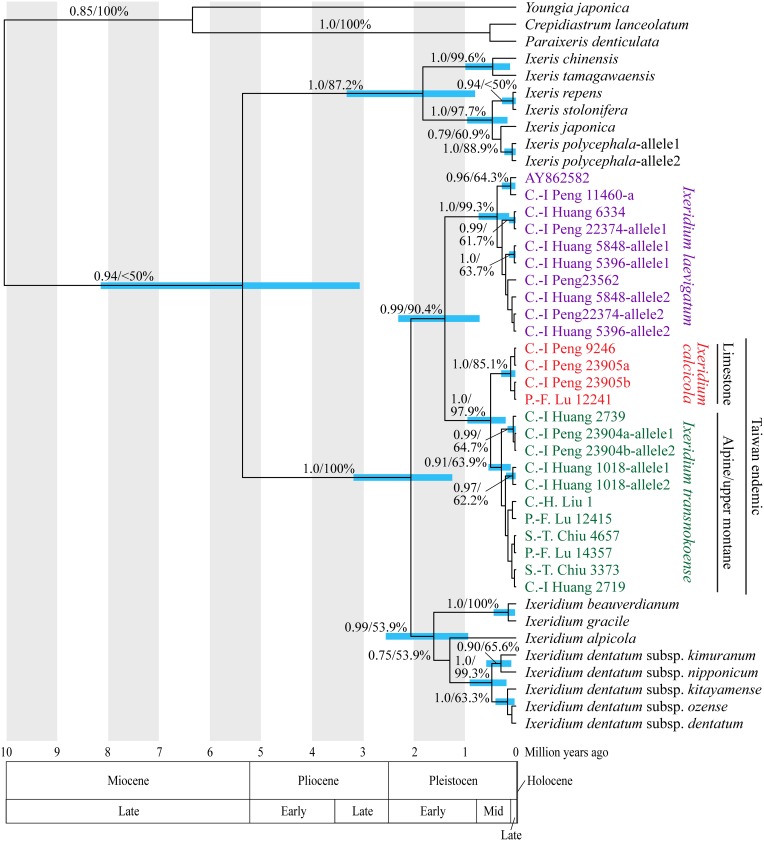
Bayesian maximum clade credibility tree of *Ixeridium*-*Ixeris*. The numerals beside branches are Bayesian posterior probabilities (PP; *left*) and bootstrap percentages (BP; *right*). Clade depth indicates mean nodal age (million years) and ingroup nodes with PP≥0.90 are annotated with the 95% highest posterior density (HPD) intervals for node ages by bars.

The aligned length of the ITS sequences was 641 bp. Two hundreds nucleotide substitutions were found in 175 variable sites and 123 sites were parsimony informative among them. The Bayesian maximum clade credibility tree is shown (Fig. 2). The MP analysis yielded the single most parsimonious tree of 272 steps with a consistency index (CI) = 0.857, a retention index (RI) = 0.945, and a rescaled consistency index (RC) = 0.810. All the clades in the most parsimonious tree (not shown) were recognized in the Bayesian tree, on which BP was plotted. Only clades supported by PP≥0.90 and/or BP≥70% are considered below. Both *Ixeridium* and *Ixeris* were recovered as monophyletic with high statistical supports (PP/BP = 1.0/100% and 1.0/87.2%, respectively). In the *Ixeridium* clade, two subclades were recovered; one comprised of the new species, *Ixeridium transnokoense*, and *Ixeridium laevigatum* (0.99/90.4%) and the other comprised of the rest of the species (0.99/53.9%). In the former subclade, the new species was sister to *Ixeridium transnokoense* (1.0/97.9%), forming a Taiwan endemic lineage. The age of the most recent common ancestor (MRCA) of *Ixeridium* and *Ixeris* was calculated as 5.37 million years ago (Ma) (95% highest posterior density [HPD] interval = 8.15–3.07 Ma). The MRCA age of the clade of the new species–*Ixeridium transnokoense*–*Ixeridium laevigatum* was estimated to be 1.39 Ma (2.31–0.71 Ma). The MRCA age of the Taiwan endemic lineage was 0.49 Ma (0.95–0.20 Ma), and that of the new species was 0.10 Ma (0.29–0.008 Ma), and that of *Ixeridium transnokoense* was 0.28 Ma (0.53–0.10 Ma).

### Chromosomal features

**Figure 3:**
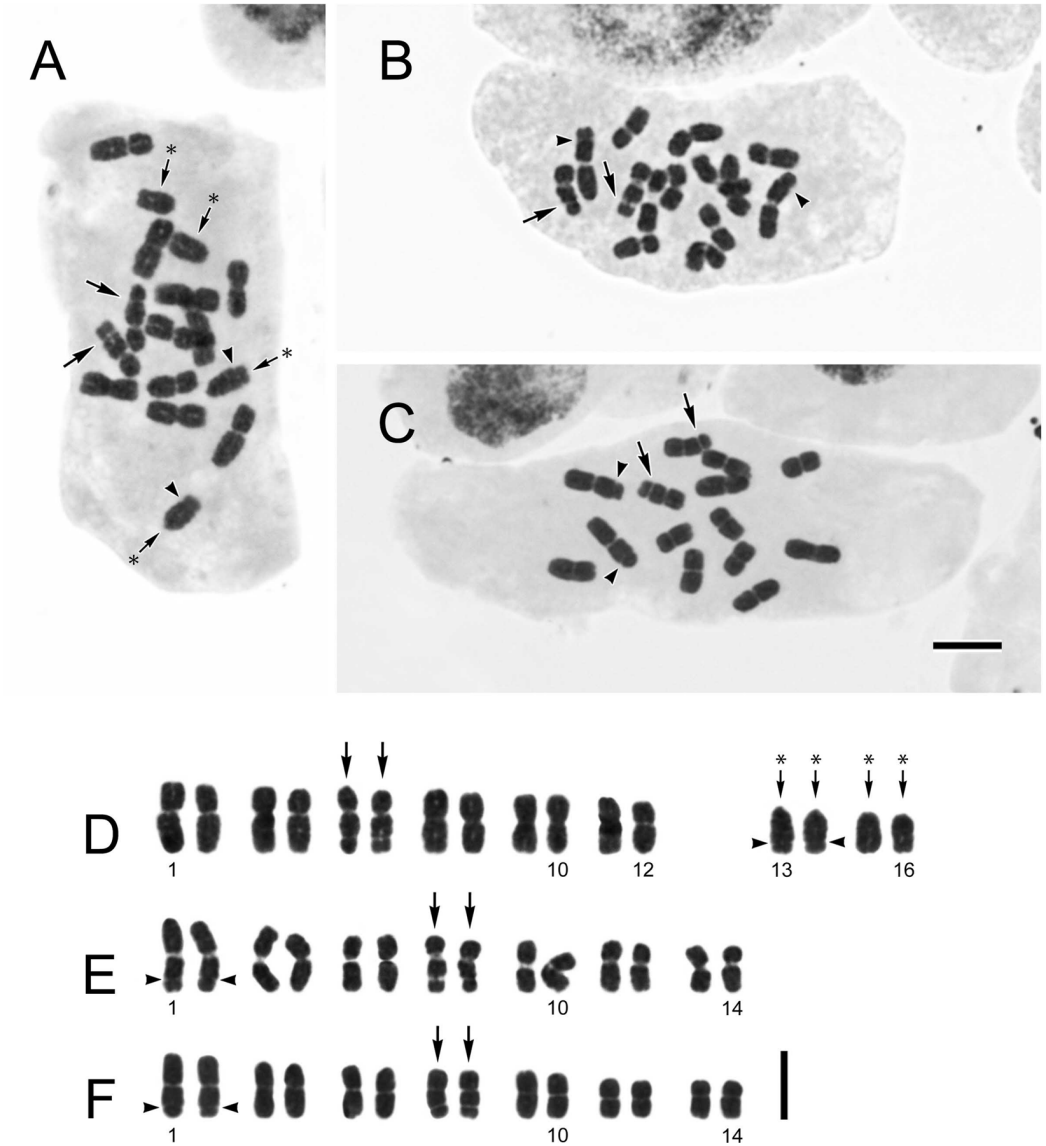
Somatic chromosomes at mitotic metaphase. A and D, *Ixeridium calcicola* (2*n* = 16); B and E, *Ixeridium laevigatum* (2*n* = 14); C and F, *Ixeridium transnokoense* (2*n* = 14). Asterisk (*) indicates t-chromosomes. Arrows indicate chromosomes with a secondary constriction (SC). Arrowheads indicate small constrictions. Scale bars, 5 ìm. Vouchers: A and D, *Ching-I Peng 23905*; B and E, *Ching-I Peng 23562*; C and F, *Ching-I Peng 23904.*

Somatic chromosome number of the new species was determined to be 2*n* = 16 (Fig. 3: A, D). Of the 16 chromosomes, four were markedly shorter than the others and had terminal (t) centromeres. The remaining 12 chromosomes varied gradually in length, eight had median (m), two had median to submedian (sm), and the other two had submedian centromeres. Secondary constrictions (SC) were observed at the interstitial region of the long arms in two submedian chromosomes (arrowed). The karyotype formula of the new species is therefore determined as 2*n* = 16 = 8 m+2 m/sm+2 sm (SC)+4 t. *Ixeridium laevigatum* (Fig. 3: B, E) and *Ixeridium transnokoense* (Fig. 3: C, F) in contrast had a somatic chromosome number of 2*n* = 14. In both, the 14 chromosomes varied gradually in length and had median centromeres. Secondary constrictions were observed at the interstitial region of the long arms in two chromosomes (arrowed). Their karyotype formula was determined as 2*n* = 14 = 14 m (2SC). Small constrictions (indicated with arrowheads) were observed at the t-chromosomes in the new species but at the longest m-chromosomes in the other two species.

### Morphological features

Detailed morphological observations found salient characters to support the placement of the new species in *Ixeridium* and to separate it from *Ixeridium transnokoense* (see Taxonomic treatment in Discussions).

## Discussion

Bayesian and MP phylogenetic analyses indicated that *Ixeridium* and *Ixeris* are phylogenetically distinct and that the new species is correctly placed in *Ixeridium*, sister to the other Taiwan endemic species *Ixeridium transnokoense*. The distinctiveness of the new species is supported by morphological, molecular phylogenetic, and cytological evidence. The new species is named *Ixeridium calcicola* C.-I Peng, S.W.Chung & T.C. Hsu (below).

### Atypical basic chromosome number

*Ixeridium calcicola* exhibited atypical chromosome number for the genus, 2*n* = 16 (Fig. 3: A, D). The basic chromosome number of *Ixeridium* is X = 7, whereas in *Ixeris* it is X = 8 [13], [14]. However, the molecular phylogenetic analyses revealed that the chromosome number of *Ixeridium calcicola* is not indicative of its placement in *Ixeris*. Small constrictions were observed at the t-chromosomes in *Ixeridium calcicola* but at the longest m-chromosomes in the other two species. This suggest that 2*n* = 16 in *Ixeridium calcicola* is derived from centric fission of the longest m-chromosomes in an ancestral karyomorphotype with 2*n* = 14. The present cytological result calls for careful observations of chromosome morphology in employing basic chromosome number as a delimiting character for *Ixeridium* and *Ixeris*.

### Phylogenetic Affinities and a limestone refugium hypothesis

The molecular phylogenetic analyses recovered the Taiwan endemic lineage comprising *Ixeridium calcicola* and *Ixeridium transnokoense* (Fig. 2). The present molecular analyses did not include *Ixeridium parvum* and *Ixeridium yunnanense* but these are less likely sister species of *Ixeridium calcicola* than *Ixeridium transnokoense* because *Ixeridium parvum* and *Ixeridium yunnanense* are local endemics more than 1,000 km apart from Taiwan [9], [18]. For the same reason, the sister relationship between the Taiwan endemic lineage and *Ixeridium laevigatum* is reliable. The MRCA age of the Taiwan endemic lineage plus *Ixeridium laevigatum* was estimated as 1.39 Ma (2.31–0.71 Ma; Fig. 2). The Taiwan endemic lineage grows in alpine/montane ranges whereas *Ixeridium laevigatum* predominantly occurs in lowlands, and the divergence is likely explained in relation to altitudinal isolation. Taiwan Island, having emerged as the Luzon arc collided with the Eurasian margins about 5 Ma, assumed the present shape at about 2 Ma through mountain building [44], [45]. The MRCA age largely postdates the mountain building events and matches the above scenario. During the Pleistocene, strong climatic oscillations occurred at regular intervals of ca. 100,000 years of cold and dry glacial periods and ca. 10,000 years of warm and moist interglacial periods [46]. In Taiwan, the temperature during glacial periods was 8.0–11.0°C cooler compared to present-day temperatures [47]. The climatic oscillation in the early Quaternary may have caused altitudinal migration and allopatric speciation between cold-tolerant lineages in alpine/mountain ranges and cold-intolerant lineages in lowlands, as was suggested for multiple plant lineages in the Northern Hemisphere [48], [49], [50], including in Taiwan [2].

Divergence time of *Ixeridium calcicola* and *Ixeridium transnokoense* was estimated as 0.49 Ma (0.95–0.20 Ma; Fig. 2). *Ixeridium calcicola* occurs at montane ranges whereas *I. transnokoense* were confined to the upper montane and alpine ranges; their divergence is also likely explained by altitudinal isolation. Their common ancestral lineage may have experienced distribution shifts between alpine and montane ranges in response to glacial-interglacial temperature fluctuation in the middle Pleistocene. A lineage which had not retreated to alpine ranges in interglacial likely survived in a limestone refugium, where ordinary plant species did not grow, leading to allopatric speciation. Dwarfism is commonly observed in calcicoles [51], [52], which may be a cause for dwelling in limestone habitats, as in this case, or a consequence of adaptation to limestone environments. Currently *Ixeridium calcicola* and *Ixeridium laevigatum* sometimes co-occurs (authors’ observation) and this is likely the result of secondarily contact after altitudinal isolation, given the above scenario. There is no edaphic barrier between the two species but no hybrid has been found. Hybridization between plants of different karyomorphotypes is largely inhibited [17], [53] and the chromosomal rearrangement in *Ixeridium calcicola* may result in a postmating barrier to gene flow, although further field survey and population genetic analyses are needed before reaching a conclusion.

### Taxonomic treatment

**Figure 4:**
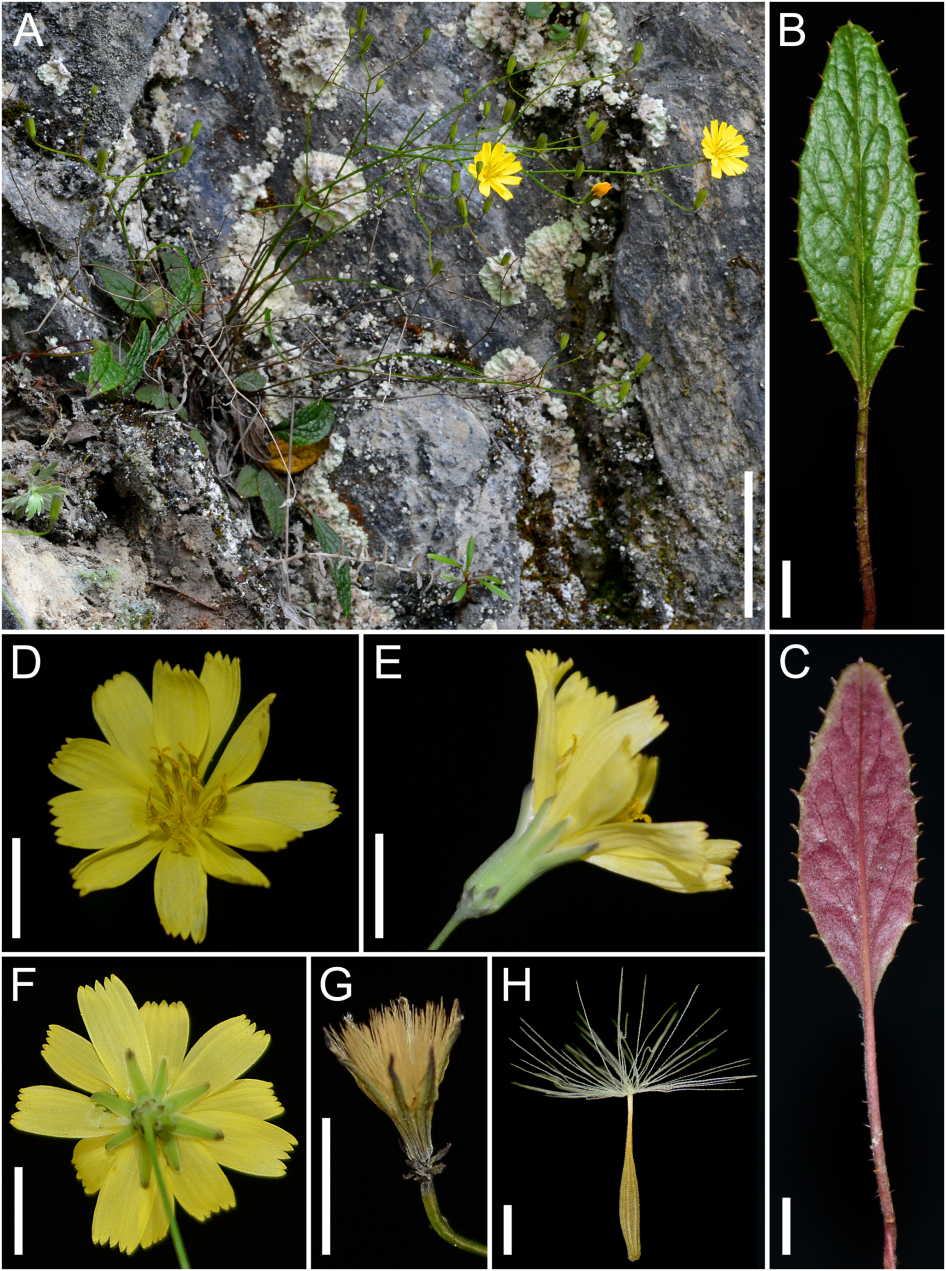
*Ixeridium calcicola*. A. Habit. B. Radical leaf, ventral view. C. Radical leaf, dorsal view. D. Capitulum. E. Involucre. F. Inner phyllaries. G. Pappus. I. Achene with pappus. Scale bars, 5 cm for A, 5 mm for B–G, 1 mm for H. All photos from *Shih-Wen Chung 11693* (HAST, TAIF, TNS).

**Figure 5:**
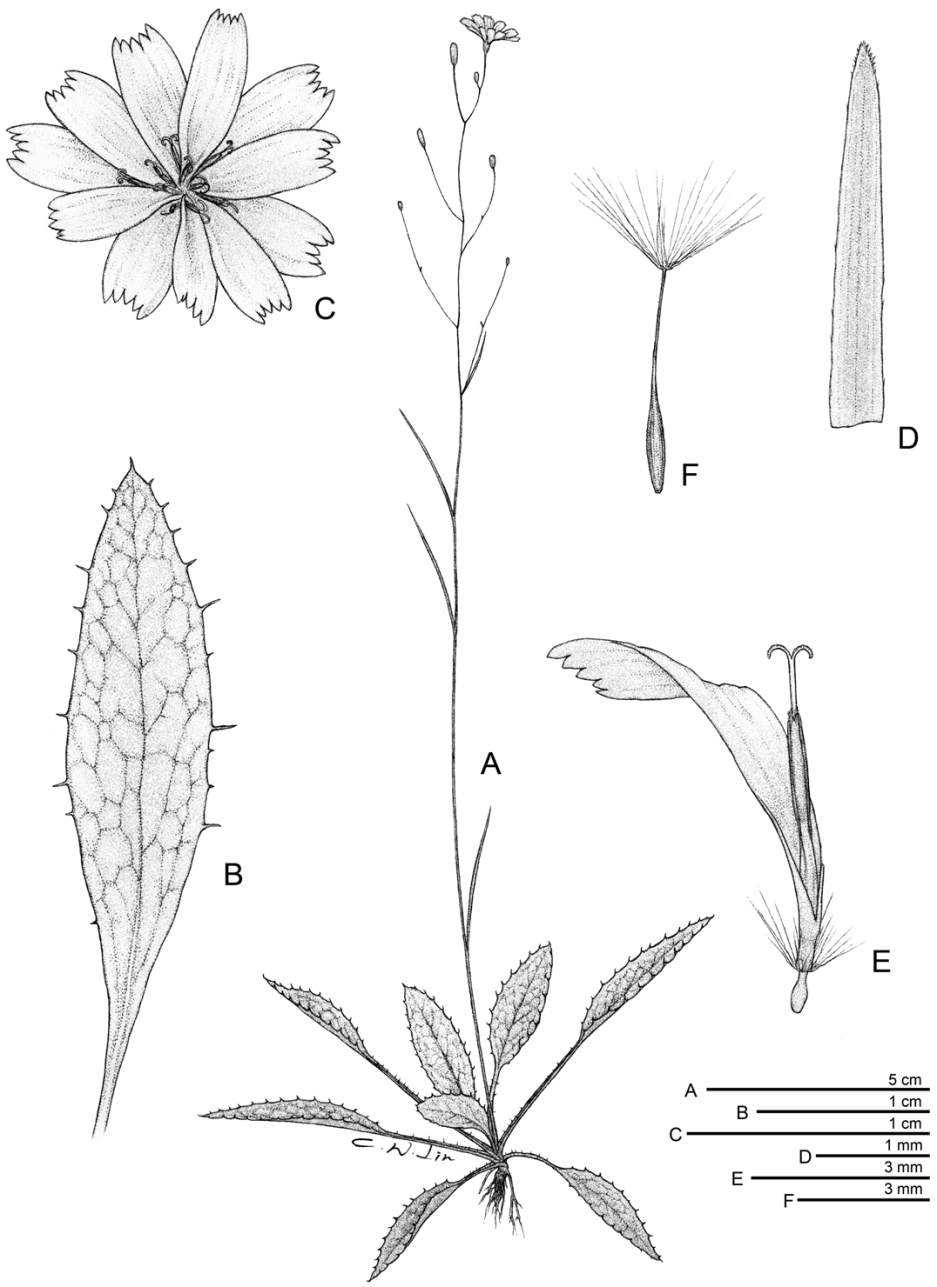
*Ixeridium calcicola*. A. Habit. B. Radical leaf blade, ventral view. C. Capitulum. D. Inner phyllary, dorsal view. E. Floret. F. Achene with pappi. All drawn from *Shih-Wen Chung 11141* (TAIF).

**Ixeridium calcicola** C.-I Peng, S.W.Chung & T.C. Hsu, sp. nov. [urn:lsid:ipni.org:names:77142104-1] (Figs. 4, 5). Type:–Taiwan. Hualien Hsien (County): Hsiulin Hsiang, Taroko National Park, On Prov. Hwy 8 (Central Cross-Island Hwy), 122.9–123 K, 24°1036.20″N, 121°2236.61″E, 2,190 m asl, 5 June 2014, *Shih-Wen Chung 11693* (holotype, TAIF; isotypes, HAST, TNS).

Rosulate, perennial herbs. Radical leaves prostrate, present at anthesis, long-petiolate; blade oblong to lanceolate, 12–54×3–16 mm, subcoriaceous, apex acute, base attenuate, glabrescent with sparse appressed fine hairs on both surfaces, green on upper surface, purple to greenish purple on lower surface, margin serrately toothed, teeth mucronulate, lateral veins 3- to 7-paired, adaxial venation sunken; petiole 8–45 mm long, sparsely covered with appressed fine hairs, purplish green to dark purple or green, margin sparsely toothed, teeth mucronulate. Cauline leaves 1 to 5; lanceolate, petiolate, 9.5–75×1–3 mm, becoming reduced, linear and subsessile along the stem. Synflorescence peduncle usually unbranched, ascending to erect, 10–30 cm tall, green, nearly-glabrous; synflorescence loosely corymbose, with 3 to ca. 20 capitula. Capitula homogamous, radiate, 15–18 mm across; peduncle capillaceous. Involucre narrowly cylindric, phyllaries abaxially glabrous; inner phyllaries 8 to 10, linear-lanceolate, 5–7 mm long, white margined, thick keeled toward base, minutely ciliolate at apex, apex acute; outer phyllaries usually 5, ovate, ca. 1.5 mm long, apex acute, calyculate. Florets (10–)11(–12), yellow, ligule 5-dentate, 8.0–9.0×3.0–4.0 mm, much exceeding involucre. Anthers 2.5–3.0 mm long, filaments 2.5–3.0 mm long, appendages ovate, apex obtuse. Style branch ca. 1.5 mm long, papillate. Achene pale brown, subfusiform, (2.5–)4–5 mm long, apex attenuate into a slender (0.5–)1–1.5 mm beak, ca. 10-ribbed, surface minutely scaly, glabrous. Pappus uniseriate, bristles many, ca 3.5 mm long, persistent, straw-colored. Somatic chromosome number, 2*n* = 16 (Fig. 3: A, D).

**Additional specimen examined:**–TAIWAN. Taiwan Island, Hualien Hsien: Terminus of the cable way at the beginning of Yenhai Forest Road, 24°0951″N, 121°3111″E, ca. 1,150 m asl, 20 July 2013, *Shih-Wen Chung 11141* (TAIF); Hoping (‘Hopin’) forest trail 41–36 K, 24°1731″N, 121°4139″E, 1,700–1,900 m asl, 25 May 1993, *S.-F. Huang 5177* (TAI); On Prov. Hwy 8 (Central Cross-Island Hwy), at Pilu, 2,000 m asl, 6 August 2006, *Pi-Fong Lu 12241* (HAST); En route from Pilushan to Pilushan Shenmu, 2,250 m asl, 5 July 1986, *Ching-I Peng 9246* (HAST); Tien-tsang Cliff, Taroko, 9 April 2008, *Ching-I Peng 23905* (HAST); Luanshan to Mt. Potolushan in the Tailuko Forestry, northwest of Hualien, 2,000–2,200 m asl, 4 August 1963, *Michio Tamura 21641* (HAST).

**Distribution, habitat, and ecology:–** *Ixeridium calcicola* is very rare, presently known from only five populations on semi-shaded to open, moist rocky ridges and cliff faces on metamorphosed limestone mountains of Taroko National Park and its vicinities in eastcentral Taiwan, ca. 1,150–2,250 m asl (Fig. 1). Flowering and fruiting season is from June to August.

**Species recognition:–** *Ixeridium calcicola* has 10 to 12 florets per capitulum, ribbed achene, and straw-colored pappus, which support its placement in the genus. *Ixeridium calcicola* has a close resemblance to *I. transnokoense* in the dwarf habit (smaller rosette and lower plant height compared with *Ixeridium laevigatum*, which grows up to 90 cm tall), but differs from the latter in the oblong to lanceolate leaf blade (vs. linear to linear-lanceolate), the mucronulate teeth on radical leaf margin and sparsely on petiole (vs. smooth to very sparse mucronulate teeth), dark purple lower leaf surface (vs. greenish), 10 to 12 florets per capitulum (vs. 5 to 7), 8 to 10 inner phyllaries (vs. 5, rarely to 7) and a basic chromosome number of X = 8 (vs X = 7).

**IUCN Red list category:–**Vulnerable (VU D2). *Ixeridium calcicola* is currently known only from a narrow area of Hualien Hsien. Although three out of five populations are located in Taroko National Park, habitat disturbance brought about by human activities such as tourism and maintenance/building of roads/walking trails may have a negative impact on the species. Additionally, the land composed mainly of deformed marble is prone to landslides especially after heavy rainfall and frequent seismic activities, which can potentially have a fatal impact on the survival of the small populations of the species.

### Key to species in Taiwan

1. Plants 10–90 cm tall; leaves usually larger than 10×1.5 cm, often pinnatilobed…………………………………. .*I. laevigatum*

1. Plants less than 30 cm tall; leaves usually smaller than 10×1.5 cm, unlobed…………………………………………….. 2

2. Leaves linear to linear-lanceolate, inconspicuously petiolate; florets 5 to 7 per capitulum; inner phyllaries 5 (rarely to 7)………………………………. *I. transnokoense*

2. Leaves oblong to lanceolate, long-petiolate; florets 10 to 12 per capitulum, inner phyllaries 8 to 10……………… *I. calcicola*

## Supporting Information

Appendix S1 ***Ixeridium transnokoense*** **specimens examined for morphological comparisons with the new species** ***Ixeridium calcicola*** **.**
(DOCX)

Appendix S2 **Species included in the molecular phylogenetic analyses.** Voucher information (only for samples sequenced in this study, which are denoted by asterisks) and GenBank accession numbers of the ITS sequences are shown.
(DOCX)
