# Supplementary material for: Ixeridium calcicola (Compositae), a New Limestone Endemic from Taiwan, with Notes on Its Atypical Basic Chromosome Number, Phylogenetic Affinities, and a Limestone Refugium Hypothesis
Source: PLoS One. 2014 Oct 8;9(10):e109797. doi: 10.1371/journal.pone.0109797 (PMC4190409; doi:10.1371/journal.pone.0109797)
Supplement: Appendix S1 — Ixeridium transnokoense specimens examined for morphological comparisons with the new species Ixeridium calcicola . (DOCX) [file pone.0109797.s001.docx]

# Appendix S1. *Ixeridium transnokoense* specimens examined for morphological comparisons with the new species *Ixeridium calcicola*.

TAIWAN. Taiwan Island, : Chilaichushannanfeng, 24°03'21"N, 121°16'39"E, 24 August 1929, *S. Sasaki s.n.* (isotype, KYO); Yushan, en route from Tatachia Saddle to Paiyun Lodge, 2,850 m asl, 31 July 1986, *Ching-I Peng 9546* (HAST); En route from Kuankao to Patungkuan, 2,650–3,000 m asl, 4 July 1985, *Ching-I Peng 8129, 8198* (HAST); Nanheng Guanshan, 24 May 1987, *Chih-Hui Chen 89* (HAST); en route from Kuankao to Chungyangchinkuang, 23°29'50"N, 120°59'50"E, 2,600–2,900 m asl, 8 July 1993, *Chih-Hsiung Chen 92* (HAST); Tienchih, 24°3'30"N, 121°14'30"E, 2,900 m asl, 16 June 1996, *Shau-Ting Chiu 3373* (HAST); Kunyang–Wuling, 24°8'10"N, 121°17'30"E, 3150 m asl, 21 July 1998, *Shau-Ting Chiu 4657* (HAST); along hiking trail, from detritus slope to Chilaipeifeng, 24°6'26"N, 121°19'51"E, 3,430 m asl, 7 November 2001, *Ya-Yi Huang 634* (HAST); near Tienchih Bridge, 23°15'52"N, 120°54'18"E, 2,350 m asl, 19 June 2002, *Chien-Hua Liu 1* (HAST); en route from hiking entrance to Kuanshanling, 23°16'7"N, 120°57'10"E, 2,900 m asl, 20 June 2002, *Chien-Hua Liu 76* (HAST); en route from Tatachia Saddle to Monroe Pavilion, above Monroe Cliff, 23°28'32"N, 120°53'52"E, 2,730 m asl, 23 July 2002, *Chien-I Huang 1018* (HAST); Nanhutashan, Taosaifeng, 24°21'35"N, 121°27'11"E, 3,450 m asl, 14 July 2002, *Wai-Chao Leong 3178* (HAST); Taroko, en route from hiking entrance to Chilai Lodge, 24°7'5"N, 121°18'44"E, 2,900 m asl, 1 July 2003, *Chien-Hua Liu 419* (HAST); Taroko, en route from Chungyangchienshan shelter to Chungyangchienshan, 24°19'8"N, 121°24'45"E, 2,950 m asl, 2 August 2006, *Chien-I Huang 2739* (HAST); Taroko, en route from Nanhu pond shelter to S-peak of Nanhutashan, 24°21'2"N, 121°25'27"E, 3,450 m asl, 1 August 2006, *Chien-I Huang 2719* (HAST); Tienchih to the hiking entrance of Nenkaoshan, 2,800–2,860 m asl, 20 August 2006, *Pi-Fong Lu 12415* (HAST); YuanFeng, 22 July 2007, *Pi-Fong Lu 14357* (HAST); YuanFeng to HsinJenKang, 2,550–2,900 m asl, 3 August 2008, *Pi-Fong Lu 16657* (HAST); Kuanshan, 23°13'52"N, 120°54'28"E, 3,000 m asl, 12 July 1935, *Noriaki Fukuyama s.n.* (TAI); Yushan, 23°28'06"N, 120°57'38"E, 2,700–3,400 m asl, 11 July 1989, *T.-C. Huang 14265* (TAI); Yunhai-Tienchih, 24°02'22"N, 121°15'07"E, 2,360–2,860 m asl, 24 August 1975, *Ho-Yih Liu 1468* (TAI); Yunlingshanchuang-Nanhupeishan-Nanhuchuanku, 24°22'12"N, 121°24'18"E, 2,450–3,530 m asl, 5 July 1986, *J.-C.Wang 3660* (TAI); Kuankao to Chunyangchinkuang, 23°30'13"N, 120°59'40"E, 2,500–2,900 m asl, 8 July 1993, *S.-F.Huang 5302* (TAI); Patungkuan-Kuankao, 23°29'50"N, 120°59'24"E, *Tokio Suzuki 13308* (TAI); Paoshan, 23°02'15"N, 120°42'42"E, 10 June 1938, *S. Sasaki 380612* (TAI); Chilaichushannanfeng, 24°03'21"N, 121°16'39"E, 24 August 1929, *Sigeyosi Suzuki 2309* (TAI); Nengkao-Yunhai, 24°02'31"N, 121°14'53"E, 25 August 1929, *Sigeyosi Suzuki 2532* (TAI); Chilaichushannanfeng, 24°03'21"N, 121°16'39"E, 4 August 1926, *Sigeyosi Suzuki s.n.* (TAI); Chilaichushannanfeng, 24°03'21"N, 121°16'39"E, 24 August 1924, *S. Sasaki 380623* (TAI); Chungyangchien, 24°18'16"N, 121°24'46"E, 3,300 m asl, 29 July 1936, *N. Fukuyama & Tokio Suzuki 15158* (TAI); Nanhuchih shelter to Nanhutashannanfong, 3,200–3,500 m asl, 7 July 2005, *Shih-Wen Chung 7881* (TAIF); Tayuling to Mt. Hohuan, 2,700–3,000 m asl, 15 June, 2011, *Shih-Wen Chung 10541* (TAIF); Kuanshanling, 3,000 m asl, 31 July 2002, *Shih-Wen Chung 5658, 5669* (TAIF); Kuanshanling, 3,100–3,200 m asl, 31 August 2002, *Shih-Wen Chung 5855* (TAIF); Yakou to Kuanshanling, 2,600–3,000 m asl, 30 July 2002, *Pei-Hsuan Lee 1894* (TAIF); Tayuling to Pilu, 2,400–2,650 m asl, 21 June 1983, *Tzer-Tong Lin s.n.* (TAIF); Mount Yiu San, 23°29'00"N, 120°54'00"E, 3,000 m asl, 5 July 1971, *Tsai-I Yan s.n.* (TAIM); Mahsiku, 27 June 1994, *Shih-Wen Chung s.n.* (TNM); 147 km point of prefectural road no. 20, between Tienchih and Guanshan, 21 June 2008, *Chih-Chiang Wang s.n.* (TNM); En route from Tienchih to boundary stone tablet, 24°02'N, 121°18'E, ca. 2,400–2,800 m asl, 21 May 1993, *Chi-Cheng Liao 1363* (TNM); Taroko, en route from Chungyangchienshan shelter to Chungyangchienshan, 24°19'8"N, 121°24'45"E, 2,950 m asl, 2 August 2006, *Chien-I Huang 2739* (TNM).
