# Supplementary material for: Ixeridium calcicola (Compositae), a New Limestone Endemic from Taiwan, with Notes on Its Atypical Basic Chromosome Number, Phylogenetic Affinities, and a Limestone Refugium Hypothesis
Source: PLoS One. 2014 Oct 8;9(10):e109797. doi: 10.1371/journal.pone.0109797 (PMC4190409; doi:10.1371/journal.pone.0109797)
Supplement: Appendix S2 — Species included in the molecular phylogenetic analyses. Voucher information (only for samples sequenced in this study, which are denoted by asterisks) and GenBank accession numbers of the ITS sequences are shown. (DOCX) [file pone.0109797.s002.docx]

# Appendix S2. Species included in the molecular phylogenetic analyses. Voucher information (only for samples sequenced in this study, which are denoted by asterisks) and GenBank accession numbers of the ITS sequences are shown.

Ingroup

*Ixeridium* (A.Gray) Tzvelev—*Ixeridium alpicola* (Takeda) Pak & Kawano, AB766225. *Ixeridium beauverdianum* (H.Lév.) Spring. (≡ *Ixeridium makinoanum* (Kitam.) Pak & Kawano), *Bai-Zhong Xiao 3555** (labelled as *Ixeris gracilis*, HAST), AB972273. *Ixeridium dentatum* (Thunb. ex Thunb.) Tzvelev subsp. dentatum, AB766214. *Ixeridium dentatum* subsp. *kimuranum* (Kitam.) Pak & Kawano, AB766223. *Ixeridium dentatum* subsp. *kitayamense* (Murata) Pak & Kawano, AB766224. *Ixeridium dentatum* subsp. *nipponicum* (Nakai) Pak & Kawano, AB766217. *Ixeridium dentatum* subsp. *ozense* (Sugim.) Yonek., AB766222. *Ixeridium gracile* (DC.) Pak & Kawano, KF154366. *Ixeridium laevigatum* (Blume) Pak & Kawano, AY862582; *Ching-I Peng23562** (HAST), AB972274; *Chien-I Huang 5848** (HAST), AB972276 (allele 1), AB972275 (allele 2); *Chien-I Huang 6334** (HAST), AB972277; *Chien-I Huang 5396** (HAST), AB972278 (allele 1), AB972279 (allele 2); *Ching-I Peng 22374** (HAST), AB972281 (allele 1), AB972280 (allele 2); *Ching-I Peng 11460-a** (HAST), AB972282. *Ixeridium calcicola* C.-I Peng, S.W.Chung & T.C. Hsu, *Ching-I Peng 23905a** (HAST), AB972283; *Ching-I Peng 23905b** (HAST), AB972284; *Pi-Fong Lu 12241** (labelled as *Ixeridium transnokoense*, HAST), AB972285; *Ching-I Peng 9246** (labelled as *Ixeris laevigata*, HAST), AB972286. *Ixeridium transnokoense* (Sasaki) Pak & Kawano, *Ching-I Peng 23904a** (HAST), AB972287; *Ching-I Peng 23904b** (HAST), AB972288; *Chien-I Huang 2719** (HAST), AB972289; *Chien-I Huang 1018** (HAST), AB972290 (allele 1), AB972291 (allele 2); *Chien-I Huang 2739** (HAST), AB972292; *Pi-Fong Lu 12415** (HAST), AB972293; *Pi-Fong Lu 14357** (HAST), AB972294; *Shau-Ting Chiu 3373** (HAST), AB972295; *Shau-Ting Chiu 4657** (HAST), AB972296; *Chien-Hua Liu 1** (HAST), AB972297. *Ixeris* (Cass.) Cass.—*Ixeris chinensis* (Thunb. ex Thunb.) Nakai, AY862578. *Ixeris japonica* (Burm.f.) Nakai (≡ *Ixeris debilis* (Thunb. ex Thunb.) A.Gray), *Chia-Hua Lin 117** (HAST), AB972298. *Ixeris polycephala* Cass., *Ching-I Peng 13667** (HAST), AB972299 (allele 1), AB972300 (allele 2). *Ixeris repens* (L.) A.Gray, HQ436225. *Ixeris stolonifera* A.Gray, AB766226. *Ixeris tamagawaensis* (Makino) Kitam., *Chi-Hsien Lin 705** (HAST), AB972301.

Outgroup

*Crepidiastrum lanceolatum* (Houtt.) Nakai, AB598563. *Paraixeris denticulata* (Houtt.) Nakai, AY876265. *Youngia japonica* (L.) DC., AB598556.
